# Numerical investigation on CFRP strengthening and reinforcement bar detailing of RC columns to resist blast load

**DOI:** 10.1016/j.heliyon.2022.e10059

**Published:** 2022-08-05

**Authors:** Tesfaye Alemu Mohammed, Solomon Abebe

**Affiliations:** a Construction Quality and Technology Center of Excellence, Addis Ababa Science and Technology University, Addis Ababa, Ethiopia; b Department of Civil Engineering, Addis Ababa Science and Technology University, Addis Ababa, Ethiopia; c Department of Civil Engineering, Debre Markos University, Ethiopia

**Keywords:** Blast load, CFRP strengthening, Rebar details, Reinforced concrete column, Scaled distance

## Abstract

The failure of column, which is a critical compressive structural member of a building, may lead to devastating progressive collapses. The present numerical study involves investigation on the performance of as-built and strengthened RC columns using 0°/90° CFRP layers under blast loading. A nonlinear FEA program, LS-DYNA was employed to study the RC columns response when subjected to blast loading. Blast field test data from recent literatures was employed for validation and further parametric studies. Variables considered were different charge masses, height of bursts, concrete compressive strengths, standoff distances, tie spacings, transverse reinforcement steel detail schemes, and 0°/90° CFRP layers. It was revealed that scaled distance parameters had a significant effect on the behaviour of RC columns under blast loading. Compared to as-built column, 0°/90° CFRP strengthened RC columns displayed excellent blast resistance. Transverse reinforcement steel details had a significant effect on lateral displacement response and failure modes of RC columns. Compared to conventionally-detailed columns, seismically-detailed columns and transverse reinforcement steel bars accompanied with square-diamond shaped ties exhibited enormous blast resistance capacity.

## 1 Introduction

Over the past decades, the need for study of explosives and respective damage mitigation techniques is becoming crucial for structural engineering world. The technology to produce powerful explosives is relatively simple and ways of delivery is as easy as parking a vehicle under a building or parking lots, or walking into a building with a briefcase or packages, and detonating explosives in a very close range or even laminating explosives to the main structural components of a building components [1]. Explosives that detonated inside parking lot of twin towers in February 1993 and intentional blast near to Alfred P. Murrah Federal Building in 1995, made members of the structural design and construction industries to be quizzed by clients about nature of blast-related hazards, risks, and methods of protections. Apparently, the need for study of blast resistant structures can be attributed to explosion related incidents still happened in the recent times. The recent observed devastating explosion event occurred at the port of the city of Beirut, the capital of Lebanon on August 2020 can be rendered as the extent to which, attention to explosions should be given.

Numerical simulation on as-built RC columns is also an effective way analyzing the blast response of structures with efficient time and computational cost. It is obvious that some data and phenomena are difficult to be observed from the experimental works can easily be accessed in numerical simulations. Various researchers performed intensive numerical simulations of FEA using LS-DYNA program to investigate the response RC columns when subjected to blast loading [2, 3, 4, 5, 6, 7, 8]. Abladey [9] used AUTODYN program to investigate the behavior of RC columns under close-in blast loads.

Zhang et al. [10] reported on a state-of-the review on blast loads induced responses of RC structural members. Collection of data in tabulated format on influence of various parameters on the blast response of RC columns, influence of different retrofitting methods on the damage mitigation of RC columns under blast loads, influence of various parameters on the blast resistance of retrofitted columns, and a summary of modelling aspects considered by previous analytical studies were presented.

An experimental investigation on the effects of close-in explosions on RC columns with different transverse reinforcement details at different scaled distances was conducted by Siba [11]. The included three test specimen column types namely: conventional, seismic and prestressed columns. Of the three columns, the prestressed columns were designed to simulates the presence of axial load in addition to own weight.

Currently, there are three major techniques for strengthening RC columns against such abnormal loading cases. (a) reinforced concrete jacketing; (b) steel jacketing; and (c) FRP confining. Among from the available techniques, when use of CFRP is compared to RC and steel jacketing techniques, its good rigidity, high ultimate strength, low density, corrosion, fatigue and vibration resistance makes CFRP sheets more and widely applicable for retrofitting of structures around the globe. Thus, to improve the strength, stiffness, durability and damage protection of RC columns, carbon fiber reinforced polymers (CFRPs) can be applied through bonding and bonding/wrapping techniques. Various researchers carried out experiments to investigate blast performance of RC square column strengthened by fibre reinforced polymers (FRPs) typically carbon fiber reinforced polymer (CFRP) under blast loading [12, 13, 14, 15, 16, 17]. All researchers reported that the use of CFRP typically improves the blast performance of RC columns by reducing both maximum and residual displacements and consequently enhancing the induced damage tolerances.

Researchers [18, 19, 20] studied performance of CFRP strengthened RC circular columns using FEA approach. FEA simulations revealed a result that CFRP strengthening could be an optimal and effective solution to mitigate the damage associated by the blast induced shock waves. Moreover, Zhang et al. [21] and Zhao et al. [22] conducted a numerical investigation on methods of enhancing capacity of RC column. Zhang et al. [21] performed a novel work to determine the effect of axial compression ratio, torsional-bending ratio, and eccentricity on the load carrying capacity of RC column strengthened by high performance ferrocement Laminate (HPFL)-bonded steel plates (BSP) prone to combined loading cases. The authors reported, all damage strengthened specimens under combined loading cases exhibited increase in load carrying capacity of specimen whereas energy absorption capacity and ductility ratios were reduced significantly. Zhao et al. [22] developed a numerical model to investigate the behavior of RC columns retrofitted with various FRPs composites such as CFRP, GFRP, and AFRP as external jacketing material for RC columns under blast loads. The authors reported among the three FRP types, CFRP performed well in enhancing blast resistance capacity of RC column. Also, from different retrofitting techniques, the authors indicated a full retrofitting mode exhibited best performance in terms of blast load resistance.

In summary, previous research studies fragmentally tried to addressed response and performance of as-built and CFRP strengthened RC columns when subjected with blast load. However, three important issues requiring further in-depth researches were recommended in previous studies [5, 9, 14]. Among recommended studies for future work on response of as-built and CFRP strengthened RC columns under blast loading includes: performing in depth parametric studies on various reinforcement schemes [5]; studying effect of various probable locations of explosives with respective to column [9]; and investigating different orientations of CFRP wraps and layer numbers [14].

Therefore, this research fills in perceived void in literature by presenting a comprehensive study on use of CFRP composite retrofitting materials including number of CFRP layers and 0°/90° CFRP orientation; on performance of RC columns with various reinforcement detailing schemes with emphasis on using various transverse reinforcement layouts including spiral reinforcements subjected to different enhanced blast load parameters and multiple blast loading scenarios. Detailed FEA procedures and results are presented in the following sections.

## 2 Finite element analysis modelling of RC column

The FE models were first generated by using a nonlinear FEA software program ANSYS-Mechanical APDL R19.0. Then by applying simulated and enhanced blast loading systems, a transient dynamic analysis was carried out using LS-DYNA FEA program.

### 2.1 RC columns details

**Figure 1:**
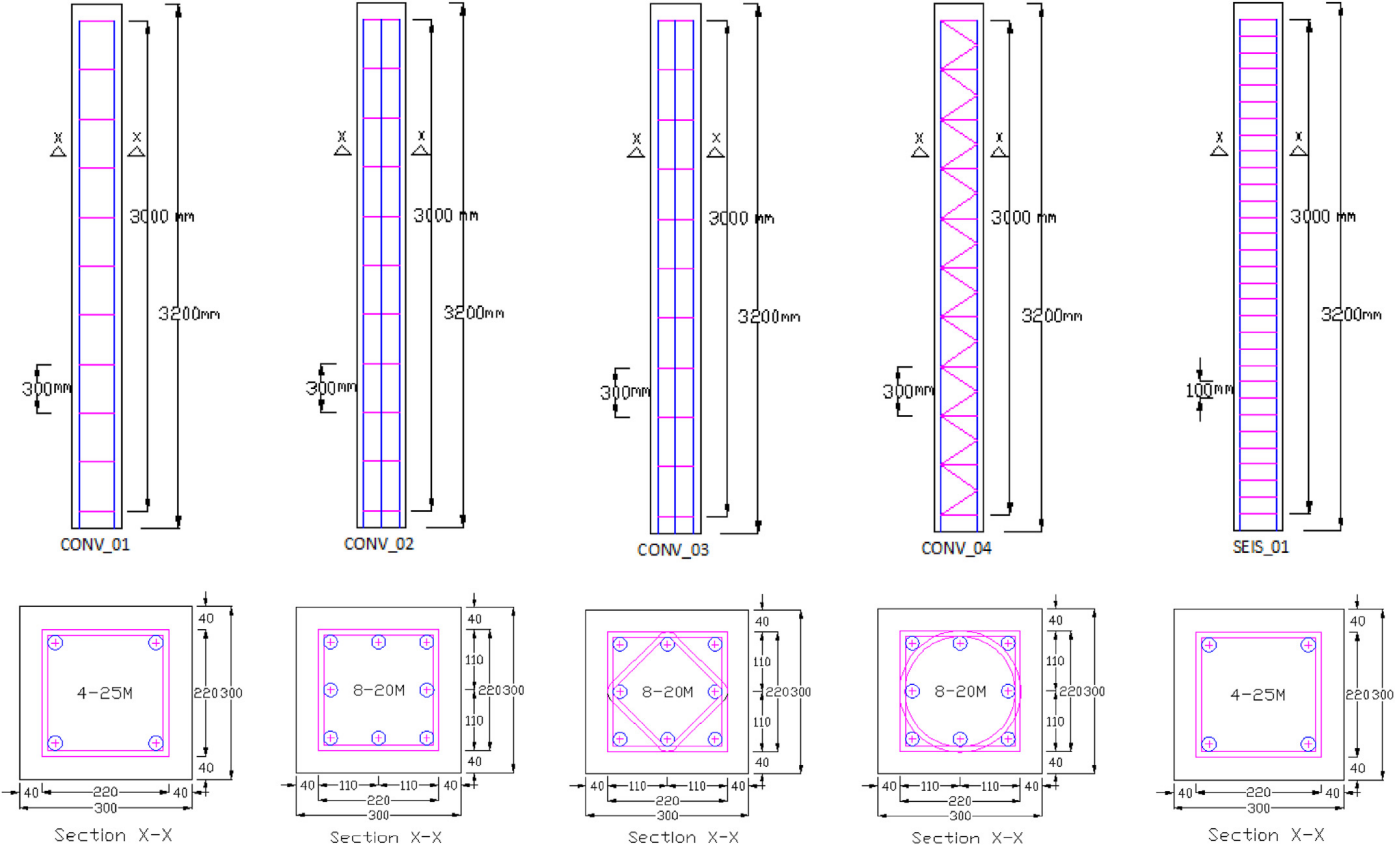
Column geometry and reinforcement details of the simulation matrix.

In this study, full scale RC column with geometric details of 300 mm × 300 mm cross section and height of 3.2 m was employed (see Figure 1). Despite geometric similarities, each RC column had different reinforcement details. RC column which was used for validation had a FEA column ID (CONV_01) or a field test column ID (CONV_07) and it was reinforced with 4–25M longitudinal bars and 10M@300 mm c/c as ties, with a concrete cover of 40 mm. The specified compressive strength of concrete was 40 MPa and the rebars had a 400 MPa tensile strength. Since column is used primarily to support an axial compressive load, a constant axial load of 1990 kN representing a real practical scenario was applied at top of a column. This load was applied as an equivalent distributed nodal load on top cross-sectional face of a RC column.

After validation, study on effect of different number of 0°/90° CFRP layers were made on CONV_01 RC column which were designed according to CSA-S806-02 [23]. CFRP strengthening was made by using FABRIC C-SHEET 240 uni-directional (UD) laminate and the CFRP material properties were taken from [24] FRP manufacturers-Italy.

Investigation on behaviour of RC columns with different transverse reinforcement steel spacings was made between two columns namely: CONV_01 and SEIS_01 (see Figure 1). CONV_01 RC column was conventionally-detailed according to clause 7.6.5.2 of [25]. Whereas, SEIS_01 was seismically-detailed according to clause 21.12.2 of [25].

Study on effect of different transverse reinforcement steel detail schemes was made between three columns namely: CONV_02, CONV_03, and CONV_04 (see Figure 1) on which they are conventionally-detailed according to clause 7.6.5.2 of [25]. The reason for the design of RC column reinforcement and CFRP layers scheme in Figure 1 is based on recommendations of CAN/CSA code of practice [23, 25] to account for accidental extreme loadings such as blast and impact loadings.

### 2.2 Finite element modeling

**Figure 2:**
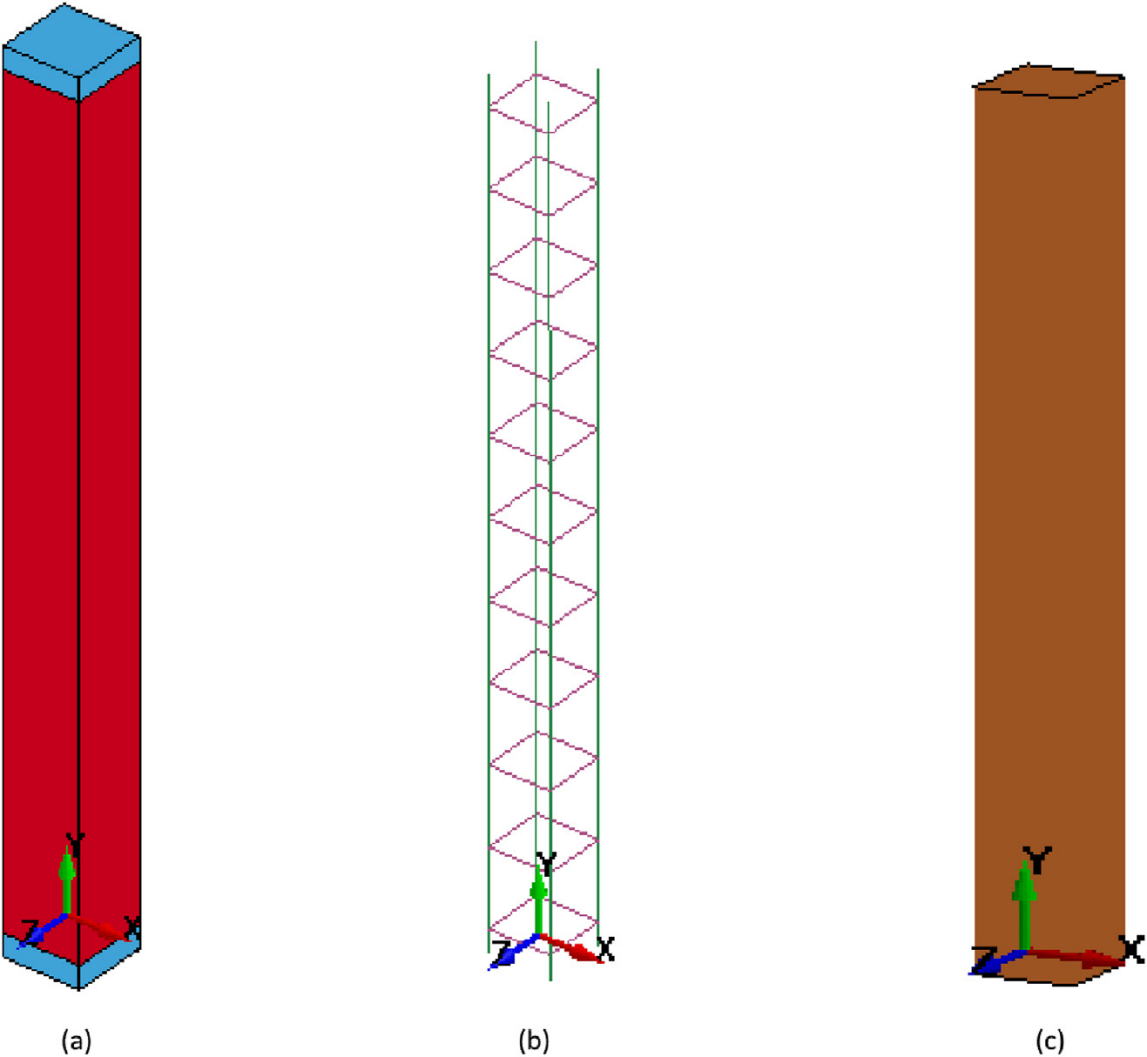
FEA model of column (CONV_01): (a) solid section; (b) beam; and (c) shell element.

LS-DYNA explicit finite element software program was used for numerical simulation and analysis. The software employs coupled-analysis modules where computational fluid dynamics (CFD) module encapsulates propagation of blast induced shock waves and computational structural mechanics (CSM) module evaluates response of structures prone to blast loads. Section geometric details and material models were defined using LS - PrePost of LS-DYNA software program. In coming sections, details of FE model generation (Figure 2) including used element and material types, boundary conditions, blast loading, Equation of State, surface contact and solution controls are presented.

### 2.3 Element type

Concrete was modelled using 8-noded hexahedron solid element with Lagrange formulation. The brick element offers to model 3-D FEA problems with high efficiency and accuracy. 30 mm mesh sizes having same aspect ratio of 1 was used in FE model generation. The solid element has a total of twenty-four degrees of freedom, three per node and the formulation of this element is done by attaching an iso-parametric (natural) coordinate system to the element. Linear displacement functions are then used to define the displacement within the solid element using nodal values. The required finite element equations are then derived using appropriate strain-displacement and stress-strain relationships [26, 27].

During element type formulation, a reduced one-point integration (a single point quadrature) with Lagrangian element formulation was employed. The biggest advantage to one-point integration is a substantial saving in computer time on which this cost saving extends to strain and element nodal force calculations where the number of multipliers is reduced by a factor of 16 when as compared to full integration (four-point) quadrature solid elements. Despite this, a one-point integration needs to control the zero-energy mode, which arise, called hourglass modes. Undesirable hourglass modes tend to have periods that are typically much shorter than the periods of the structural responses, and they are often observed to be oscillatory. One way of resisting undesirable hourglassing is with a viscous damping or small elastic stiffness capable of stopping the formation of anomalous modes but having a negligible effect on the stable global modes [26, 27].

Steel rebar reinforcements were idealized using modified beam element supporting only uniaxial loads. All longitudinal and transverse rebars were discretized with 30mm element size identical with concrete elements. A steel plate with 300 mm × 300 mm cross sectional dimension and 90 mm thickness were used for end plates at top and bottom of the column. This use support and loading plates enable uniform stress distribution of applied axial load thus reducing stress concentration effect. The end plates were modeled with 8 node brick element and discretized with 30 mm mesh size having aspect ratio of 1. Moreover, CFRP composites were modeled by using a four-node membrane (shell) element on which the element has compatible degree of freedom with the constant solid element. Equal spacing integration points and 0°/90° CFRP fibre orientation were used in modeling of CFRP composites. CFRP composites was discretized with mesh size of 30mm and aspect ration of 1. In present study, we have used Lagrangian method as such effect of air medium was not included.

### 2.4 Material model

LS-DYNA material library offers wide range of material and equation of state (EOS) models, each with a unique number of input variables. In the present study, ∗MAT_159_CONTINOUS_SURFACE_CAP_MODEL (CSCM) material model which is specifically designed for predicting response of concrete material under blast loads was employed. The model has smooth or continuum surface cap model and is available for use with solid elements. CSCM material model has been used to analyze dynamic loading of RC structures [26, 27, 28]. The parameters of concrete used in the validation section are listed in Table 1.

**Table 1:** Summary of concrete material properties.

Property	Designation	Value	Unit measure
Cylinder strength	$f\prime_{c}$	40	MPa
Tension Strength	$f_{t}$	1.97	MPa
Peak strain	$e_{o}$	2.10	mm/m
Elastic modulus	$E_{c}$	32000	MPa
Mass density	ρ	2.7 × 10^−9^	tonne/mm^3^
Poison ratio	ν	0.2	--

Steel reinforcement is used in RC structures to provide the tensile strength that concrete lacks. In present study, LS-DYNA material model ∗MAT_003R3_ELASTIC_PLASTIC_WITH_KINEMATIC_ISOTROPIC was used to characterize steel rebar material properties. The model has efficient way of modelling of strain rate effect. Linear isotropic hardening capability was used for both longitudinal and transverse steel rebar. Table 2 illustrates the material property for both longitudinal and transverse reinforcement steel.

**Table 2:** Summary of reinforcement steel material properties.

Property	Designation	Value	Unit measure
Yield strength	$f_{y}$	400	MPa
Ultimate strength	$f_{u}$	600	MPa
e-strain harden	$\varepsilon_{sh}$	7	mm/m
Rupture strain	$\varepsilon_{u}$	100	mm/m
Elastic modulus	$E_{s}$	200000	MPa
Mass density	ρ	7.8 × 10^−9^	tonne/mm^3^
Poison ratio	ν	0.3	--

CFRP composite was modeled by using ∗MAT_002_TROPIC_ELASTIC element type on which this material is valid for modelling the elasto-orthotropic behaviour of solids, shells and thick shells. CFRP material data was taken from [24] FRP manufacturers-Italy. The parameters of CFRP used for the analysis are presented in Table 3.

**Table 3:** CFRP composite material properties.

Property	Designation	Value	Unit measure
Specific gravity	$G_{s}$	1.8	g/cm^3^
Weight of the fabric	w	600	g/m^2^
Thickness	t	0.334	mm
Elastic modulus	$E_{s}$	240	GPa
Tensile strength	ft	3800	MPa
Mass density	ρ	1.0 × 10^−12^	tonne/mm^3^
Poison ratio	ν	0.1	--

### 2.5 Boundary conditions

Single Point Constraint (∗SPC) system that constraints both translation and rotation on top and bottom ends of the column was utilized throughout the analysis. This all-support condition was implemented by using a typical value of 1 in ∗SPC_SET keyword command on which input value of 1 restraint nodal displacements (translation and rotation).

### 2.6 Loading

Three loading conditions namely self-body-weight, axial and blast loads are imposed into the system by using the ∗LOAD_BODY, ∗LOAD_BEAM and ∗LOAD_BLAST_ENHANCED keyword files, respectively. Self-weight of a column is taken into account by using ∗LOAD_BODY command. It requires two input variables namely curve id (LCID) and gravity scale factor (SF = -0.00981) applied through ramp load step system whereas axial load was uniformly applied at top of a RC column using ∗LOAD_BEAM command. The command prompts use of node id (NSID), direction of applied load (DOI), specified load curve id (LCID) and scale factor (SF = -1).

Blast loading protocol is defined by employing ∗LOAD_BLAST_ENHANCED keyword command. The command prompts user to define explosive's charge center (XBO, YBO and ZBO), unique blast id (BID), type of blast source (BLAST) and unique segment set id representing a face of column where ∗LOAD_BLAST_ENHANCED command applies generated blast wave pressures. Moreover, blast wave parameters such as charge mass and standoff distance were entered into CONWEP to evaluate time duration in terms of positive and negatives phase out. This time duration was entered into LS-DYNA to define termination in blast load analysis. Blast induced shock wave strikes front face of a RC column on YZ plane to induce deformation in X-direction.

### 2.7 Surface contact

An Automatic-General and General-Transducer-Penalty algorithm-based interaction models were utilized for creating a bond between concrete and reinforcement steel bars. A slave and master definitions for reinforcement steel and concrete elements were assigned respectively. By doing so, the reinforcement steel bars and concrete elements share nodes to achieve a fully coupled interaction (perfect bond) with no slip. Moreover, perfect bond was assumed between CFRP and concrete. This is enforced by using ∗AUTOMATIC_ SURFACE_TO_SURFACE_TIEBREAK contact algorithm.

### 2.8 Strain rate effects

Response of structures against extreme loading such as blast and impact loading require consideration of strain rate effect. In the present study, strain rate effect was considered by activating LS-DYNA ∗ IRATE command keyword. This command enables to capture dynamic properties of materials and respective damage propagations subjected to blast loading.

### 2.9 Equation of state

The Equation of state (EOS) employed in this study was LS-DYNA's ∗EOS_LINEAR_ POLYNOMIAL which easily characterizes nature of a very high strain rate and material pressures far in excess of yield stress, and propagation of shock waves. By doing so, material models that require an accompanying EOS calculate deviatoric state of stress including strength behavior and pressure components of total stress (hydrostatic behavior).

### 2.10 Solution controls

The solution controls including energy, hourglass, termination time and time step keywords were defined to create a stable solution on energy control keyword catalogue. An hourglass energy and energy dissipation were computed and included in the energy balance. For hourglass viscosity type a typical standard type, specifically a standard LS-DYNA value with 0.1 hourglass coefficient which makes the dynamic blast load analysis stable was used. The initial time step size was then permitted to be determined by LS-DYNA analysis module.

### 2.11 Displacement monitoring points (gauges)

The measure of displacement at a given point was captured by using a specific monitoring point (gauges). Nodal displacements were analyzed using ASCII_NODOUT file command with three monitoring points (gauges) located at lower one-third (1000 mm), middle (1600 mm), and upper one-third (2100 mm) along the length of column.

### 2.12 Database

Nodal displacement values for a given blast scenario were interpreted using American Standard Code for Information Interchange (ASCII) NODOUT file command. Whereas, the damage profile was extracted from the plastic strains of fringe components.

## 3 Finite element analysis results

### 3.1 Validation of FE model

It is obvious that, FEA is an efficient numerical method for solving different type of engineering problems with complicated geometries, loadings and material properties. This all happen if the mathematical and numerical model definitions are good enough. One means of checking the reliability of the numerical model is conducting validation of numerical FE models by field controlled experimental data. In this study, a typical RC column specimen with field test column ID (CONV_07) designed by [11] was used for validation purpose.

During validation process, the total run time of mesh sizes smaller than 30 mm, made a substantial increase in the computing time, leads the risk of memory overflow, an additional memory to complete solutions, an additional dynamically allocated memory and additional number of CPU have been required by the operating system. In order to achieve maximum computing efficiency thereby reducing run-time without distracting the numerical computational accuracy, a 30 mm mesh sizes is allowed to be used throughout the analysis.

**Figure 3:**
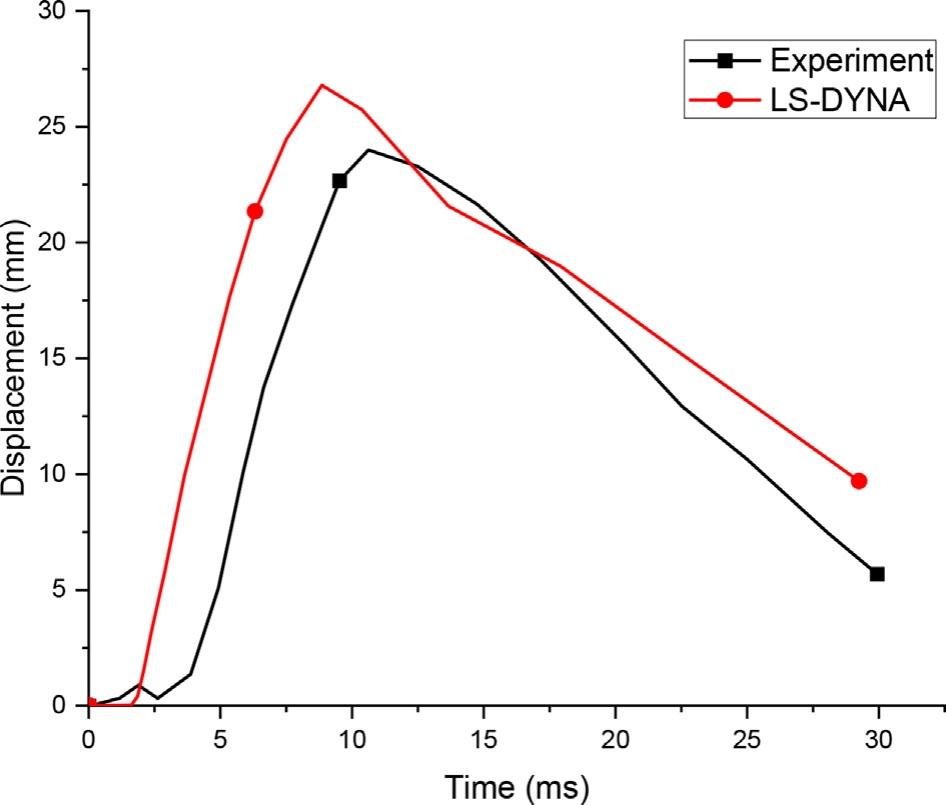
Displacement time history curves for experimental and FEA.

The post-blast field test result revealed that, the maximum displacement of 24.00 mm occurred at lower one-third along the height of the column measured from the bottom support. Whereas, the FEA depicted a maximum displacement of 26.8 mm located on lower one-third along the height of column measured from bottom support. Figure 3 elucidates comparison of FEA displacement time history curves and experimental work.

**Figure 4:**
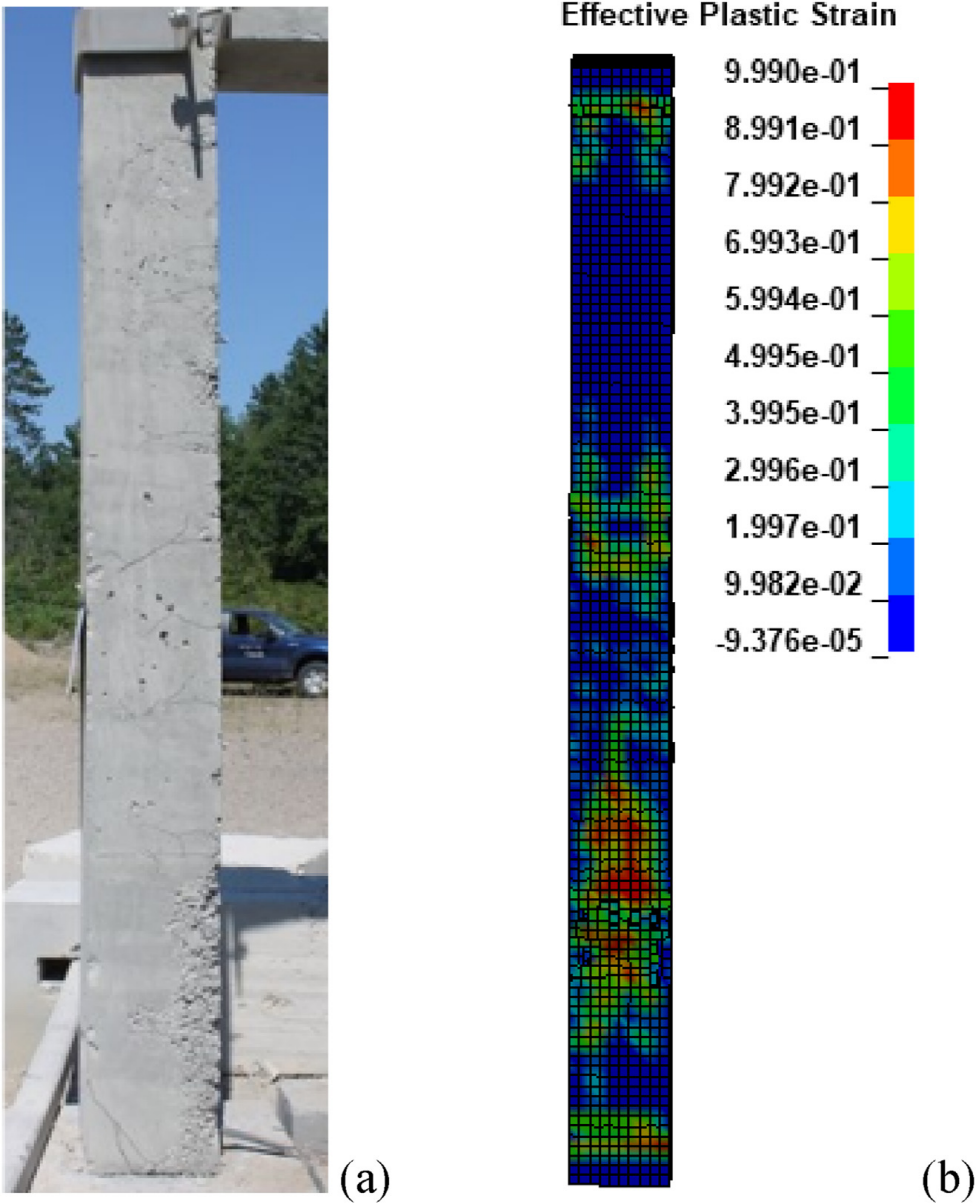
Post-blast column damages: (a) experiment [11]; and (b) FEA.

Figure 4 (a) and (b) illustrates post-blast column damages for one of the specimens tested in the experiment (CONV_07) and corresponding FE model (CONV_01). Figure 4 (a) shows post-test column damage on the column. Physical observation on selected RC column CONV_07 after the live field blast impact showed substantial cracking and spalling of concrete cover revealing longitudinal reinforcement in the lower one-third region of the column [11]. As shown in Figure 4 (a) minimal flexural cracks were observed on the back-face of the column. Within the lower one-third region, substantial shear cracks were also observed. As shown in Figure 4 (b) LS-DYNA numerical simulation after blast impact shows a first strike, maximum displacement and respective damage at the lower one-third region of the column as well.

## 4 Parametric study of RC columns under blast loading

### 4.1 Numerical simulation matrices

**Figure 5:**
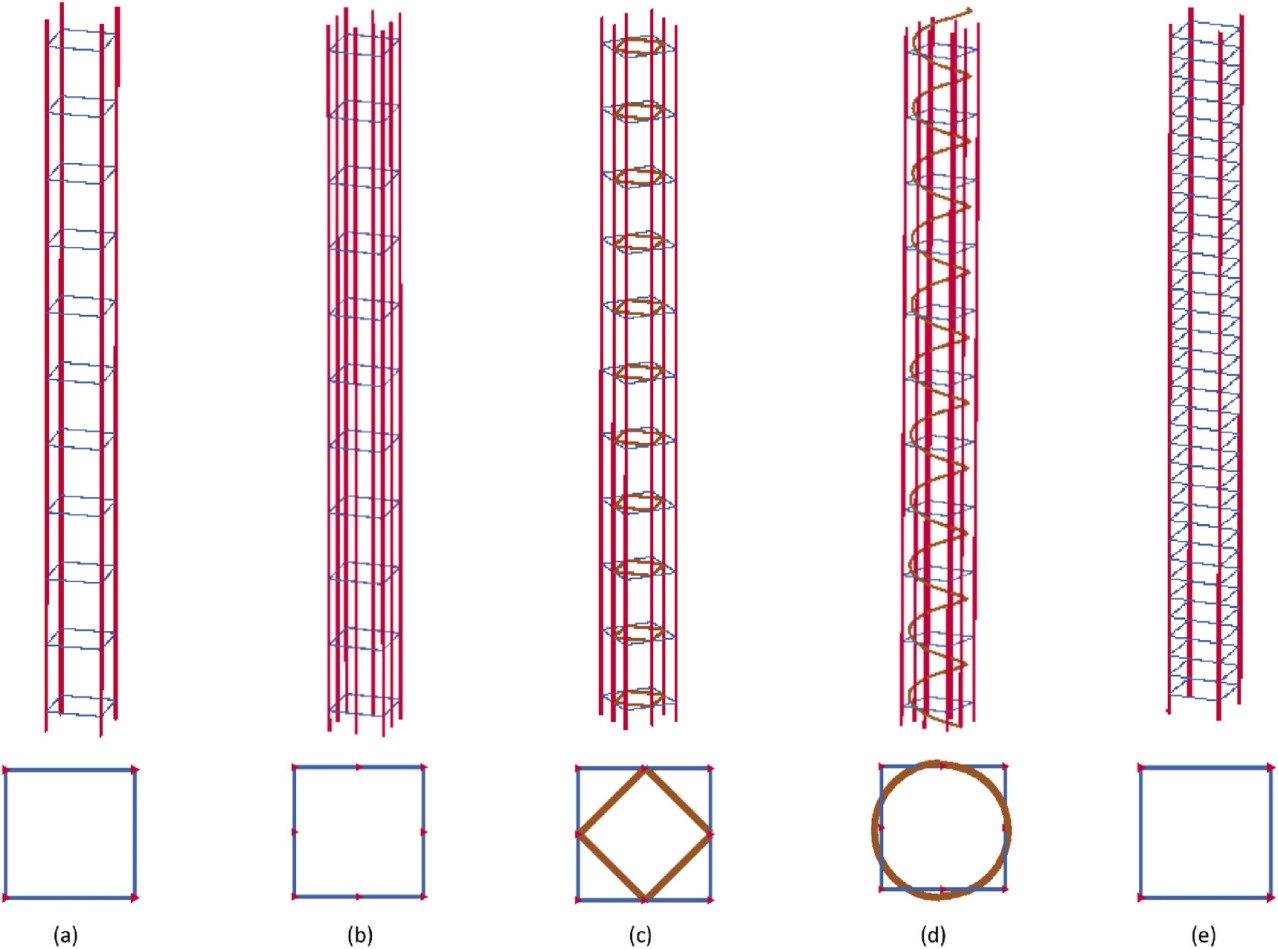
LS-DYNA FEA models: (a) CONV_01; (b) CONV_02; (c) CONV_03; (d) CONV_04; (e) SEIS_01.

A parametric analysis was conducted after the generated FE model was validated with the field controlled recent experimental tests results, then numerous parameters including lateral displacement response of RC columns under the effects of different charge masses (CHM), standoff distances (SOD), height of bursts (HOB), grades of concrete (GOC), and different reinforcement details (transverse steel reinforcement spacings and detail schemes) with respective enhanced blast load positive and negative time durations (t_d_^+^ & t_d_^−^) are studied. The reason to have this reinforcement detail schemes is to investigate various configurations and enable a comprehensive RC column reinforcement detail schemes provided in [25]. As presented in Table 4, to determine the influence of each parameter, they were discussed by varying a single parameter while keeping other parameters constant. Correspondingly, the performance of 0°/90° CFRP strengthened RC columns were investigated in the parametric section. Figure 5 illustrates the FEA models developed for the parametric study. The columns shown in Figure 5 consists of five RC column used in the parametric study, with same cross section 300 mm × 300 mm and height of 3.2 m. Despite this similarity, all columns had different reinforcement ratio, transverse reinforcement spacings and detail schemes.

**Table 4:** Summary for simulation matrix and respective blast scenarios.

Effect of different explosive charge masses on CONV_01 RC column								
Blast scenario	CHM (kg)	Blast source	GOC	HOB (m)	Reinforcement grade	SOD (m)	t_d_^+^ (ms)	t_d_^−^ (ms)
1A	50	Surface burst	M40	1.6	S400	5	8.1	47.7
2A	100	Surface burst	M40	1.6	S400	5	9.3	58.0
3A	250	Surface burst	M40	1.6	S400	5	5.0	73.7
4A	400	Surface burst	M40	1.6	S400	5	3.7	84.8
Effect of different concrete compressive strength on CONV_01 RC column								
Blast scenario	GOC	Blast source	CHM (kg)	HOB (m)	Reinforcement grade	SOD (m)	t_d_^+^ (ms)	t_d_^−^ (ms)
1B	M20	Surface burst	400	1.6	S400	5	3.7	84.8
2B	M30	Surface burst	400	1.6	S400	5	9.3	58.0
3B	M40	Surface burst	400	1.6	S400	5	5.0	73.7
4B	M50	Surface burst	400	1.6	S400	5	3.7	84.8
Effect of different height of bursts on CONV_01 RC column								
Blast scenario	HOB (m)	Blast source	CHM (kg)	GOC	Reinforcement grade	SOD (m)	t_d_^+^ (ms)	t_d_^−^ (ms)
1C	0	Surface burst	250	M40	S400	3	1.7	70.4
2C	0.6	Surface burst	250	M40	S400	3	1.7	70.4
3C	1.6	Free-air burst	250	M40	S400	3	1.7	70.4
4C	3.2	Free-air burst	250	M40	S400	3	1.7	70.4
Effect of different standoff distances on CONV_01 RC column								
Blast scenario	SOD (m)	Blast source	CHM (kg)	GOC	HOB (m)	Reinforcement grade	t_d_^+^ (ms)	t_d_^−^ (ms)
1D	3	Surface burst	100	M40	1.6	S400	2.1	53.2
2D	5	Surface burst	100	M40	1.6	S400	9.3	58.0
3D	7	Surface burst	100	M40	1.6	S400	10.0	61.3
4D	10	Surface burst	100	M40	1.6	S400	9.7	62.9

Effect of different transverse reinforcement steel spacings on CONV_01 RC column									
Blast scenario	Z $\left(\frac{m}{kg^{\frac{1}{3}}}\right)$	Blast source	CHM (kg)	GOC	HOB (m)	Reinforcement grade	SOD (m)	t_d_^+^ (ms)	t_d_^−^ (ms)
1E	0.61	Surface burst	70	M40	0	S400	2.5	1.6	47.0
2E	0.89	Surface burst	130	M40	0	S400	4.5	11.11	60.8
3E	1.00	Surface burst	50	M40	0	S400	3.7	3.24	45.6
4E	0.61	Free-air burst	70	M40	1.6	S400	2.5	1.6	47.0
5E	0.89	Free-air burst	130	M40	1.6	S400	4.5	11.11	60.8
6E	1.00	Free-air burst	50	M40	1.6	S400	3.7	3.24	45.6
Effect of different transverse reinforcement steel spacings on SEIS_01 RC column									
1F	0.61	Surface burst	70	M40	0	S400	2.5	1.6	47.0
2F	0.89	Surface burst	130	M40	0	S400	4.5	11.11	60.8
3F	1.00	Surface burst	50	M40	0	S400	3.7	3.24	45.6
4F	0.61	Free-air burst	70	M40	1.6	S400	2.5	1.6	47.0
5F	0.89	Free-air burst	130	M40	1.6	S400	4.5	11.11	60.8
6F	1.00	Free-air burst	50	M40	1.6	S400	3.7	3.24	45.6
Effect of different transverse reinforcement steel schemes on CONV_02 RC column									
1G	0.35	Surface burst	200	M40	0	S400	2.05	1.3	64.2
2G	0.65	Surface burst	100	M40	0	S400	3.00	2.1	53.2
3G	0.85	Surface burst	150	M40	0	S400	4.5	5.2	63.1
4G	1.00	Surface burst	125	M40	0	S400	5.00	4.42	61.8
Effect of different transverse reinforcement steel schemes on CONV_03 RC column									
1H	0.35	Surface burst	200	M40	0	S400	2.05	1.3	64.2
2H	0.65	Surface burst	100	M40	0	S400	3.00	2.1	53.2
3H	0.85	Surface burst	150	M40	0	S400	4.5	5.2	63.1
4H	1.00	Surface burst	125	M40	0	S400	5.00	4.42	61.8
Effect of different transverse reinforcement steel schemes on CONV_04 RC column									
1I	0.35	Surface burst	200	M40	0	S400	2.05	1.3	64.2
2I	0.65	Surface burst	100	M40	0	S400	3.00	2.1	53.2
3I	0.85	Surface burst	150	M40	0	S400	4.5	5.2	63.1
4I	1.00	Surface burst	125	M40	0	S400	5.00	4.42	61.8

It is worthy to note design of parameters and the way enhanced blast load generation were chosen in accordance with vulnerability assessment of [29] on which available scaled distances in the parametric study designated critical standoff distances in uncontrolled area of a given perimeter (see also section 2.3 of [29]). In addition to this, type of burst followed in the parametric study was also designed in accordance with predictions of section 4.1.3 of [29]. Hemispherical surface and spherical air burst types also simulate type of blast effects prediction for a given structure based on a typical luggage bomb to a large truck bomb detonated in and above the ground respectively.

In general, the choice of parameters including enhanced blast load parameters (SOD, HOB, CHM, Td), blast load type (hemispherical surface load and spherical air burst) whether luggage based or truck-based explosion events, and type of reinforcement detail schemes were diligently selected in accordance with design manuals and guidelines [25, 29].

To determine the improvement on use of 0°/90° CFRP composites, the following expression was used,

(1) $$\Delta_{decrment.}\left(\text{\%}\right)=\left(\frac{\Delta_{CFRP\;strengthened}-\Delta_{as-built}\;}{\Delta_{as-built}}\right)x100$$

Where Δ_decrement._ (%) = lateral displacement decrement (%), Δ_CFRP_ strengthened = lateral displacement of CFRP strengthened RC column, & Δ_as-built_ = lateral displacement of as-built RC column.

## 5 Numerical results of RC column under blast loading

### 5.1 Effect of different explosive charge masses

To study the effect of explosive charge masses, four scaled distances Z $\left(\frac{m}{kg^{\frac{1}{3}}}\right)$ of 1.36, 1.08, 0.79, and 0.68 were considered. At each of these scaled distances, the RC columns, were subjected to blast loading from charge mass (kg) of 50, 100, 250, and 400.

**Figure 6:**
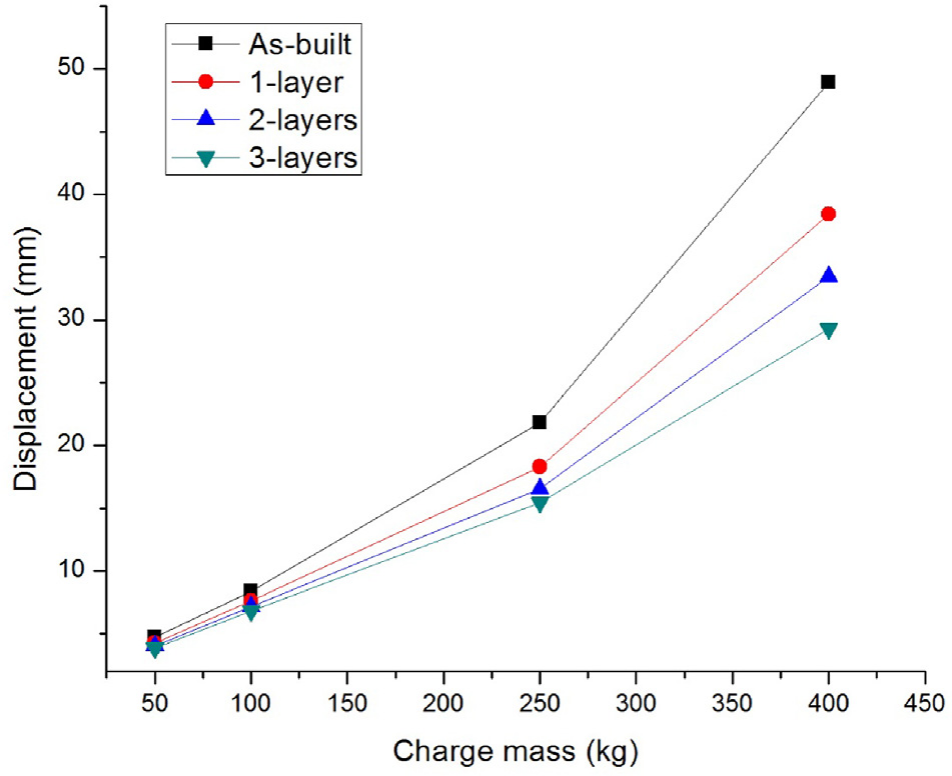
Comparison of lateral displacement versus various charge masses for as-built and CFRP strengthened RC columns.

As shown in Figure 6, higher magnitude of charge masses produces longer blast loading durations and large lateral displacements. When the explosive CHM (kg) increased from 50 to 400, an increase in lateral displacement (mm) ranging from 4.7 to 48.91, 4.24 to 38.42, 4.01 to 33.48, and 3.85 to 29.26 were observed for as built-bare and strengthened columns with 1-layer, 2-layers, and 3-layers of 0°/90° CFRP wraps respectively.

Compared to as-built column, as number of 0°/90° CFRP layers increased from 1 to 3, the strengthened columns' lateral displacement was dropped (%) considerably up to 18.09, 18.28, 29.16, and 40.18 for blast scenario 1A, 2A, 3A, and 4A respectively (see Table 5). This suggests that the effect of CFRP wrap to minimize blast damage, is more critical for a structural system with higher explosive charge masses.

**Table 5:** Improvement in lateral displacement response of CFRP strengthened as compared to as-built RC columns for different charge masses.

Number of CFRP layers	Charge mass (kg)			
	50	100	250	400
	Lateral displacement decrements (%)			
1	9.79	9.08	16.09	21.45
2	14.68	15.17	24.26	31.55
3	18.09	18.28	29.16	40.18

### 5.2 Effect of different concrete compressive strengths

To study the effect of concrete compressive strength on column responses under blast loading, standard concrete grades of M20, M30, M40, and M50 were considered. The scaled distance Z was kept at 0.68 $\frac{m}{kg^{\frac{1}{3}}}\;$ for all column models.

**Figure 7:**
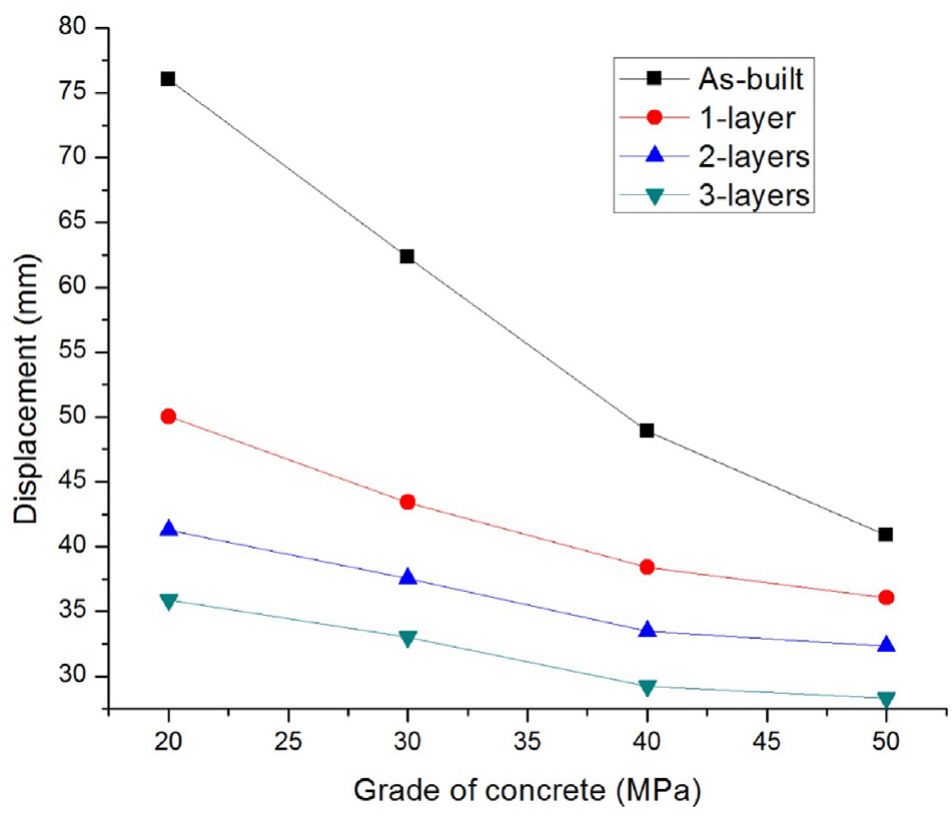
Comparison of lateral displacement versus various concrete grades for as-built and CFRP strengthened RC columns.

As shown in Figure 7 with an increase in the concrete grades from M20 to M50, the fall of lateral displacement (mm) ranging from 76.03 to 40.89, 50.03 to 36.05, 41.25 to 32.31, and 35.89 to 28.33 were observed for as-built and strengthened columns with 1-layer, 2-layers, and 3-layers of 0°/90° CFRP wraps respectively.

Compared to as-built column, as number of 0°/90° CFRP layers increased from 1 to 3, the strengthened columns' lateral displacement was dropped (%) significantly up to 52.79, 46.99, 40.24, and 30.72 for blast scenario 1B, 2B, 3B, and 4B respectively (see Table 6). This suggests that the effect of CFRP wrap to minimize blast damage, is more significant for a structural system with low-grade of concrete.

**Table 6:** Improvement in lateral displacement response of CFRP strengthened as compared to as-built RC columns for different concrete compressive strength.

Number of CFRP layers	Grade of concrete			
	M20	M30	M40	M50
	Lateral displacement decrements (%)			
1	34.20	30.27	21.45	11.84
2	45.75	39.63	31.55	20.98
3	52.79	46.99	40.24	30.72

### 5.3 Effect of different height of bursts

To study the effect of height of bursts, four scaled distances Z $\left(\frac{m}{kg^{\frac{1}{3}}}\right)$ of 0.54, 0.50, 0.48, and 0.54 and a 250 kg TNT CHM having height of burst (m) of 0, 0.6, 1.6, and 3.25 were considered.

**Figure 8:**
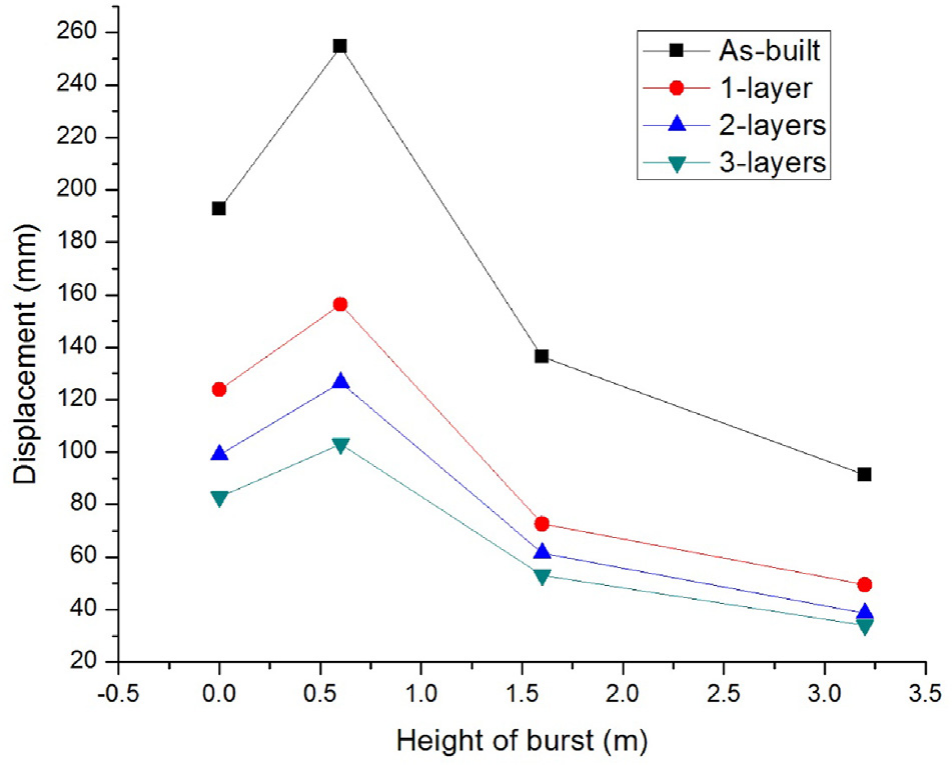
Comparison of lateral displacement versus various height of bursts for as-built and CFRP strengthened RC columns.

As shown in Figure 8, the higher magnitude of explosives generated from a ground type of burst would produce a large amount of impulse leading to large lateral displacements. As the blast type changed from spherical free air burst to hemispherical surface burst and HOB (m) coordinate decreased from 3.2 to 0, an increase in the lateral displacements (mm) ranging from 91.45 to 254.74, 49.45 to 156.37, 38.70 to 126.56, and 33.87 to 103.2 were observed for as-built and CFRP strengthened columns with 1- layer, 2-layers, and 3-layers of 0°/90° CFRP wraps respectively.

Compared to as-built column, as number of 0°/90° CFRP layers increased from 1 to 3 in the strengthened columns' lateral displacements fall (%) considerably up to 56.98, 59.49, 61.10, and 62.96 for blast scenario 1C, 2C, 3C, and 4C respectively (see Table 7).

**Table 7:** Improvement in lateral displacement response of CFRP strengthened as compared to as-built RC columns for different height of burst.

Number of CFRP layers	Height of burst (m)			
	0	0.6	1.6	3.2
	Lateral displacement decrements (%)			
1	35.73	38.62	46.74	45.93
2	48.66	50.32	54.94	57.68
3	56.98	59.49	61.10	62.96

### 5.4 Effect of different standoff distances

To study the effect of stand-off distances, four scaled distances Z $\left(\frac{m}{kg^{\frac{1}{3}}}\right)$ of 0.65, 1.08, 1.51, and 2.15 were considered. At each of these scaled distances, the RC columns, were subjected to blast loading from standoff distances (m) of 3, 5, 7, and 10 SODs.

**Figure 9:**
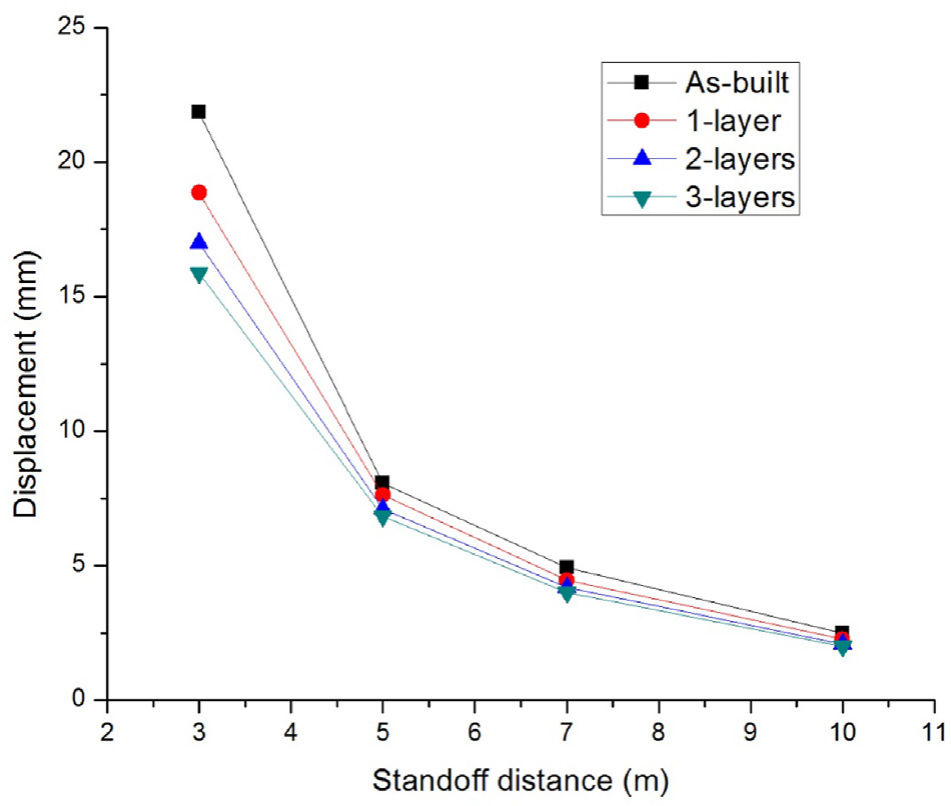
Comparison of lateral displacement versus various standoff distances for as-built and CFRP strengthened RC columns.

Figure 9 shows that as SOD (m) increases from 3 to 10, the lateral displacement decreased exponentially from 21.86 to 2.50, 18.83 to 2.26, 17 to 2.09, and 15.89 to 1.99 for as-built and CFRP strengthened columns with 1-layer, 2-layers, and 3-layers of 0°/90° CFRP wraps respectively.

Compared to as-built column, as number of 0°/90° CFRP layers increased from 1 to 3 in the strengthened columns' lateral displacement was dropped (%) significantly up to 27.36, 15.03, 19.11, and 20.40 for blast scenario 1D, 2D, 3D, and 4D respectively (see Table 8). This suggest that the effect of CFRP wrap to minimize blast damage, is more crucial for a structural system with small standoff distances.

**Table 8:** Improvement in lateral displacement response of CFRP strengthened as compared to as-built RC columns for different standoff distances.

Number of CFRP layers	Standoff distance (m)			
	3	5	7	10
	Lateral displacement decrements (%)			
1	13.86	5.34	9.35	9.60
2	22.23	11.80	14.84	16.40
3	27.36	15.03	19.11	20.40

### 5.5 Effect of different transverse reinforcement steel spacings

**Figure 10:**
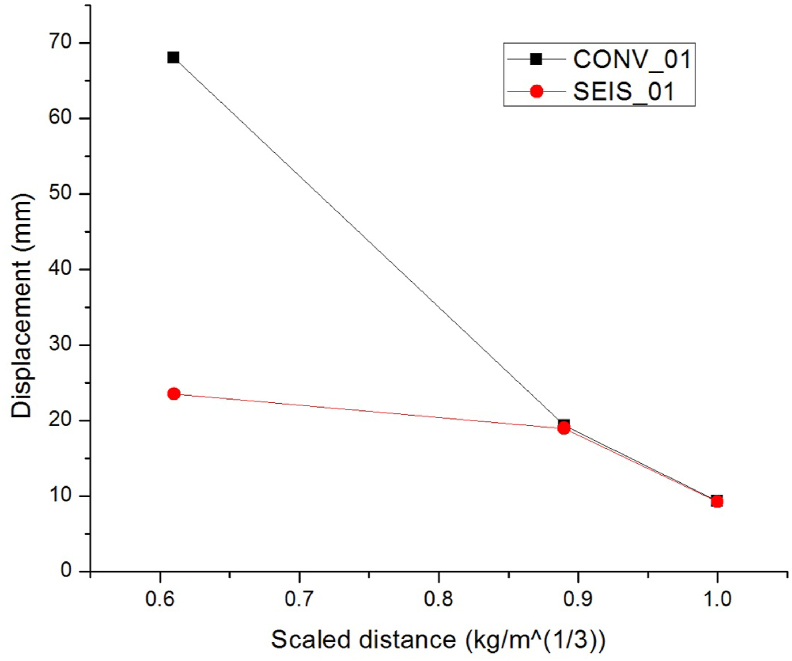
Comparison of lateral displacement vs scaled distances of CONV_01 and SEIS_01 RC columns for hemispherical surface bursts.

**Figure 11:**
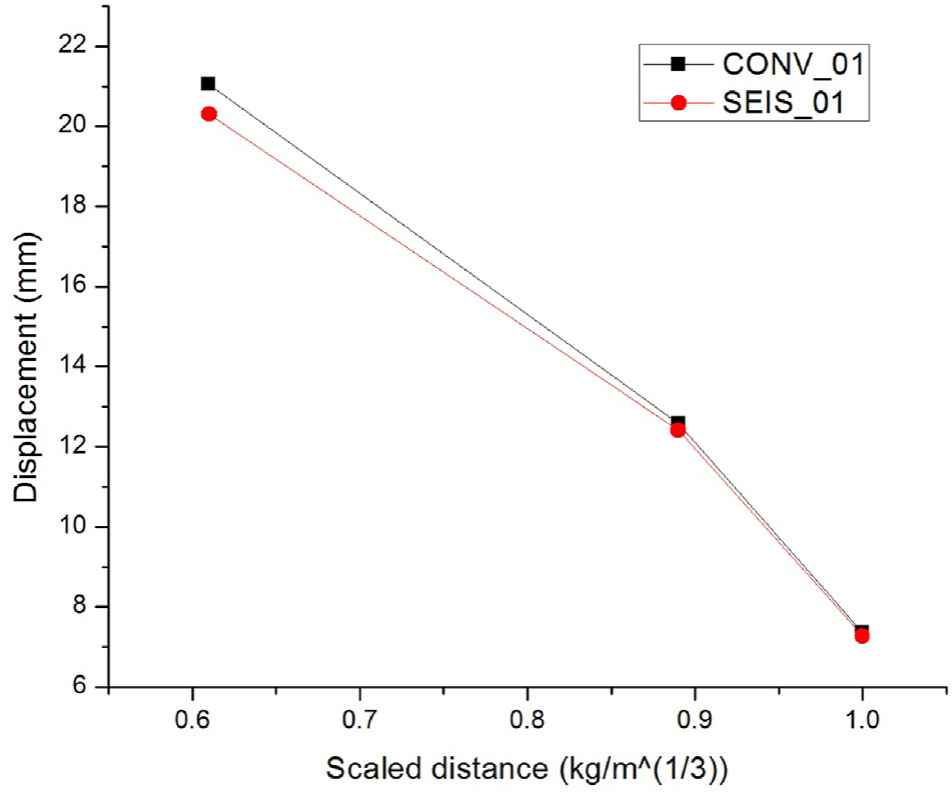
Comparison of lateral displacement vs scaled distances of CONV_01 and SEIS_01 RC columns for spherical free air bursts.

To study the effect of spacing of transverse reinforcements on RC column response three scaled distances Z $\left(\frac{m}{kg^{\frac{1}{3}}}\right)$ of 0.61, 0.89 and 1.0 on CONV_01 & SEIS_01 RC columns were considered. Figures 10 and 11 illustrates lateral displacement values for CONV_01 and SEIS_01 RC column for a given blast scenarios with hemispherical surface and spherical free-air bursts respectively, and from Figures 10 and 11, SEIS_01 RC column typically for hemispherical surface bursts revealed significant fall in displacement values when as compared to CONV_01 RC columns.

Table 9 illustrates the lateral displacement decrement (%) values of two RC columns (CONV_01 and SEIS_01) with three scaled distances Z $\left(\frac{m}{kg^{\frac{1}{3}}}\right)$ typically 0.61, 0.89 and 1.00 simulating close-in blast events accompanied by hemispherical surface bursts on which the initial blast induced shock waves are amplified by the ground. Compared to CONV_01 RC column, SEIS_01 RC column's lateral displacements dropped (%) up to 65.38, 1.93, and 0.69 for blast scenario 1E, 2E, 3E, 1F, 2F and 3F respectively (see Table 9). This suggests that minimizing tie spacings is more critical for small scaled distance blast scenarios.

**Table 9:** Improvement in lateral displacement response of seismically-detailed as compared to conventionally-detailed RC column for different scaled distances under hemispherical surface bursts.

Scaled distance Z $\left(\frac{m}{kg^{\frac{1}{3}}}\right)$	Lateral displacement decrements (%)
0.61	65.38
0.89	1.93
1.00	0.69

Table 10 shows the lateral displacement difference (%) values of two RC columns (CONV_01 and SEIS_01) with three scaled distances Z $\left(\frac{m}{kg^{\frac{1}{3}}}\right)$ namely 0.61, 0.89 and 1.00 simulating close-in blast events accompanied by spherical free air bursts on which the initial blast induced shock waves are not amplified by the ground. Compared to CONV_01 RC column, SEIS_01 RC column's lateral displacements decreased with minimal value (%) up to 3.53, 1.26, and 1.18 for blast scenario 4E, 5E, 6E, 4F, 5F and 6F respectively (see Table 10). This suggests that minimizing tie spacings is not much important for spherical free-air burst types.

**Table 10:** Improvement in lateral displacement response of seismically-detailed as compared to conventionally-detailed RC column for different scaled distances under spherical free-air bursts.

Scaled distance Z $\left(\frac{m}{kg^{\frac{1}{3}}}\right)$	Lateral displacement decrements (%)
0.61	3.53
0.89	1.26
1.00	1.18

**Figure 12:**
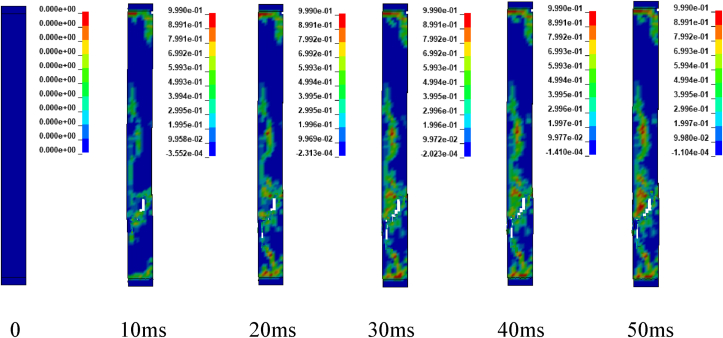
Effective plastic contour of CONV_01 RC column for blast scenario-2E.

**Figure 13:**
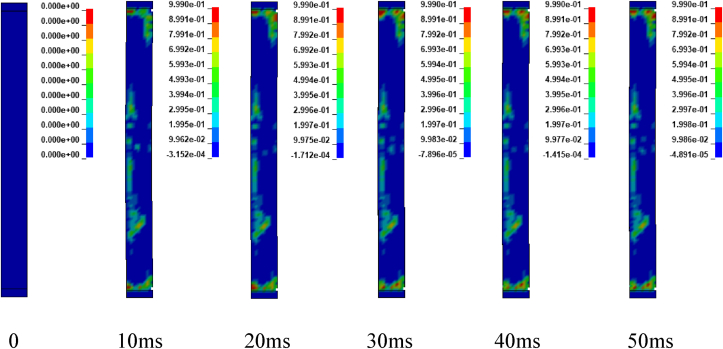
Effective plastic contour of SEIS_01 RC column for blast scenario-2F.

Figures 12 and 13 illustrates sample damage model for CONV_01 and SEIS_01 columns under blast scenario of 2E, 2F, 4E and 4F accompanied by hemispherical surface bursts. The damage threshold values were extracted from effective plastic strains fringe component modules. Figure 12 shows, CONV_01 column damage and the concrete in the bottom part of the column was severely destroyed and dilated leaving portion of the column. Within less than 5 ms, the failure progression extends from the bottom to the half part of the column. Direct shear failure type was observed on the location where the column was restrained. The rationale behind this type of failure is due to presence of blast induced shock wave in small scaled distance ranges $\left(\text{Z}\leq 1\frac{m}{kg^{\frac{1}{3}}}\right)$. Reinforcement bars were deformed (bent) in the bottom part of the column. On the other hand, SEIS_01 depicts minor damage in the mid height of the column. Minor direct shear failure type was observed on the top and bottom location where the constraints were applied. A very slight failure progression started to spread after 10 ms (see Figure 13).

**Figure 14:**
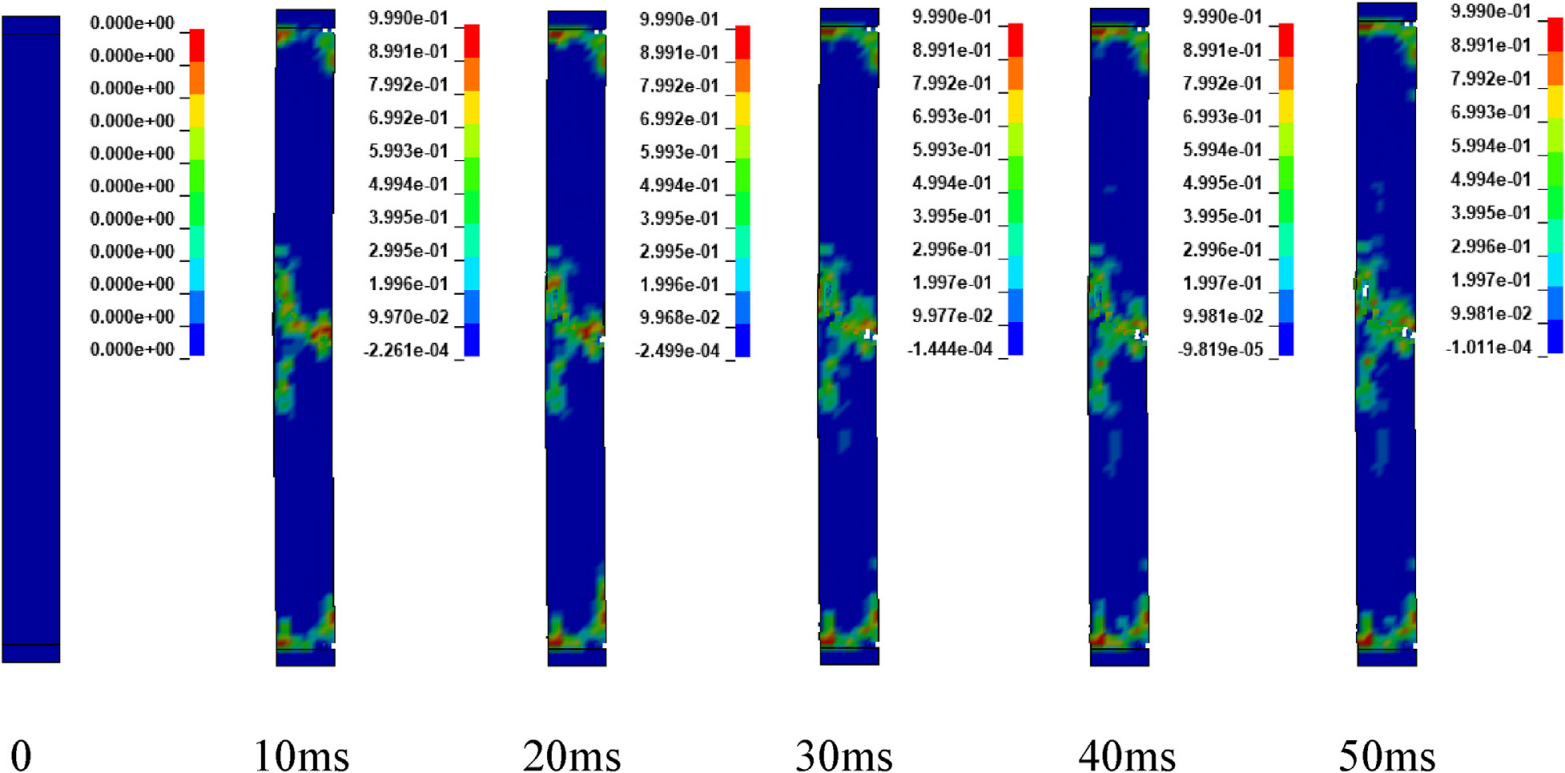
Effective plastic contour of CONV_01 RC column for blast scenario-4E.

**Figure 15:**
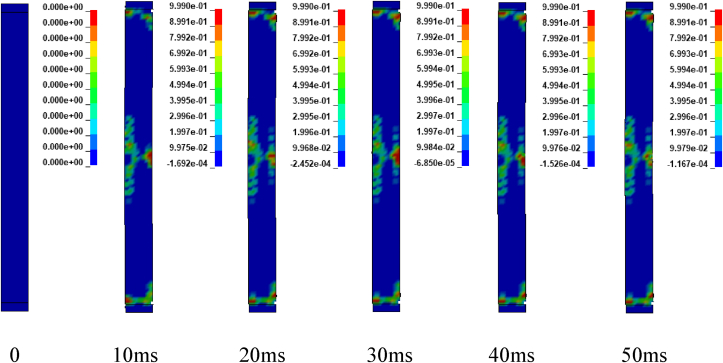
Effective plastic contour of SEIS_01 RC column for blast scenario-4F.

Figures 14 and 15 represents sample damage model for CONV_01 and SEIS_01 columns under blast scenario of 2E, 2F, 4E and 4F accompanied by spherical free-air bursts. Figure 14 shows, CONV_01 column damage and the concrete in the mid height of the column where the explosive was located, was destroyed and dilated leaving portion of the column. Minor direct shear failure type was observed on the top and bottom locations where constraints were applied. Reinforcement bars were deformed (bent) in the mid height of the column. While, SEIS_01 column depicts minor damage in the mid height of the. Minor direct shear failure type was observed on the location where the column was restrained when as compared (see Figure 15).

### 5.6 Effect of different transverse reinforcement steel detail schemes

To study the effect of different transverse reinforcement steel detail schemes on RC column response, four scaled distances Z $\left(\frac{m}{kg^{\frac{1}{3}}}\right)$ of 0.35, 0.65, 0.85 and 1.00 on CONV_02, CONV_03 & CONV_04 RC columns were considered.

**Figure 16:**
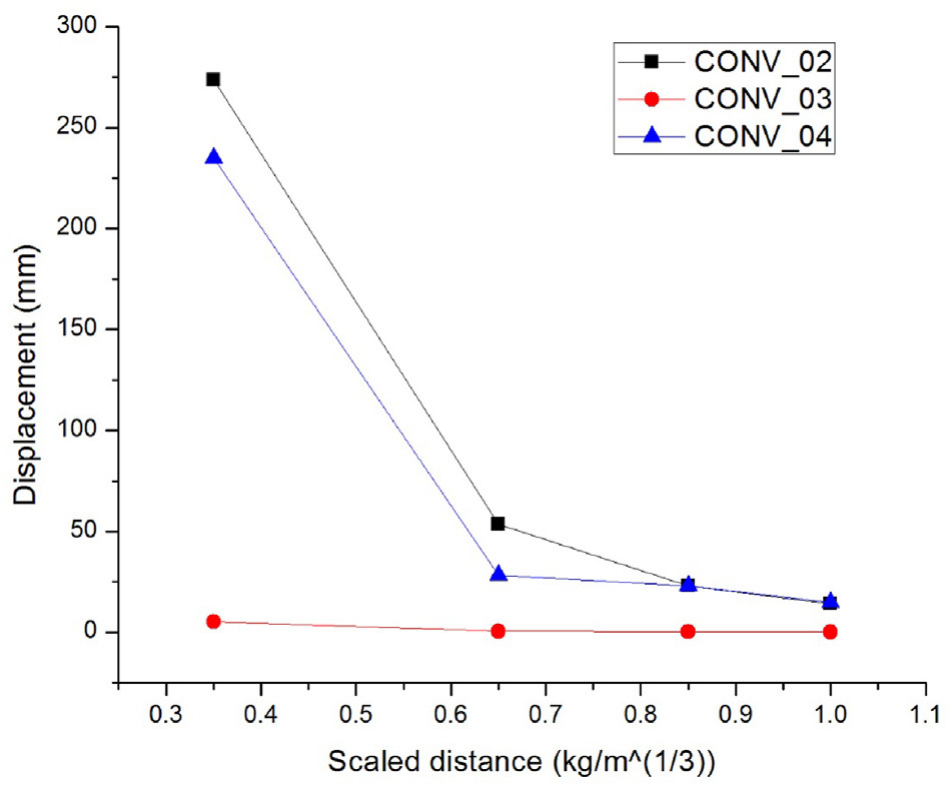
Comparisons of lateral displacement vs scaled distances for CONV_02, CONV_03 and CONV_04 RC columns with hemispherical surface bursts.

Figure 16 depicts that different type of transverse steel reinforcement detail schemes from the square to square-diamond and square-spiral shaped ties, a substantial decrease in displacement was obtained, especially for one of the details in CONV_03 column which was accompanied by square-diamond ties exhibits remarkable performance under close-in blast scenarios. In other words, a CONV_03 RC column with well concrete core confinement revealed an excellent performance over CONV_02 and CONV_04 RC column under close-in blast loads.

Compared to CONV_02 column, CONV_03 RC column elucidates significant falls in lateral displacement (%) up to 98.10, 99.07, 98.89, and 98.92. On the hand CONV_04 RC column following CONV_03 column, depicts second most improvement in lateral displacement decrements (%) up to 14.09, 47.25, 0.76, and 3.94 (see Table 11). This suggests that, due to excellent concrete confinement mode, a typical transverse reinforcement steel detail scheme (square-diamond shaped tie) is more vital for hemispherical surface burst type blast scenarios.

**Table 11:** Improvement in lateral displacement response of diamond and spiral shaped transverse reinforcement steel detail schemes as compared to square tie shaped RC columns.

RC column ID.	Scaled distance Z $\left(\frac{m}{kg^{\frac{1}{3}}}\right)$			
	0.35	0.65	0.85	1.00
	Lateral displacement decrements (%)			
Improvement in lateral displacement of CONV_03 when as compared to CONV_02 RC columns	98.10	99.07	98.89	98.92
Improvement in lateral displacement of CONV_04 when as compared to CONV_02 RC columns	14.09	47.25	0.76	3.94

**Figure 17:**
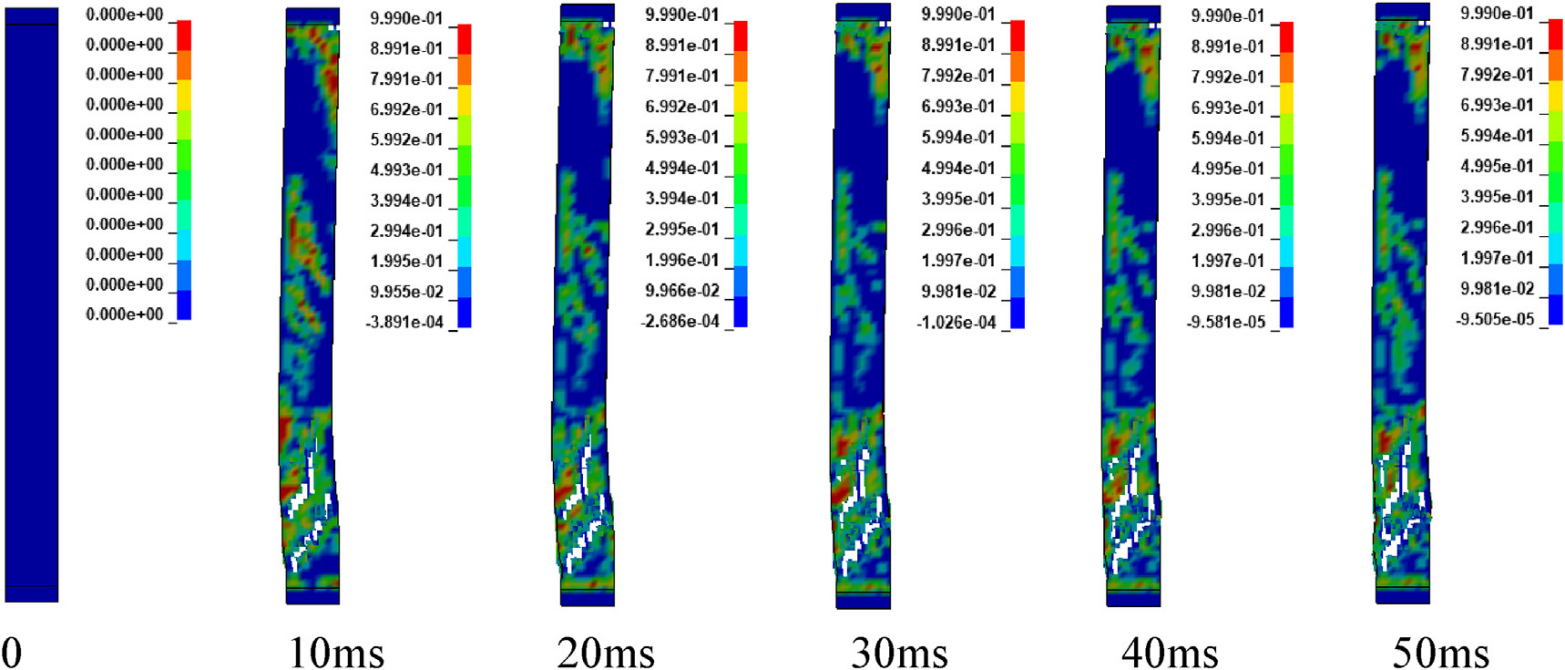
Effective plastic contour of CONV_02 RC column for blast scenario-2G.

**Figure 18:**
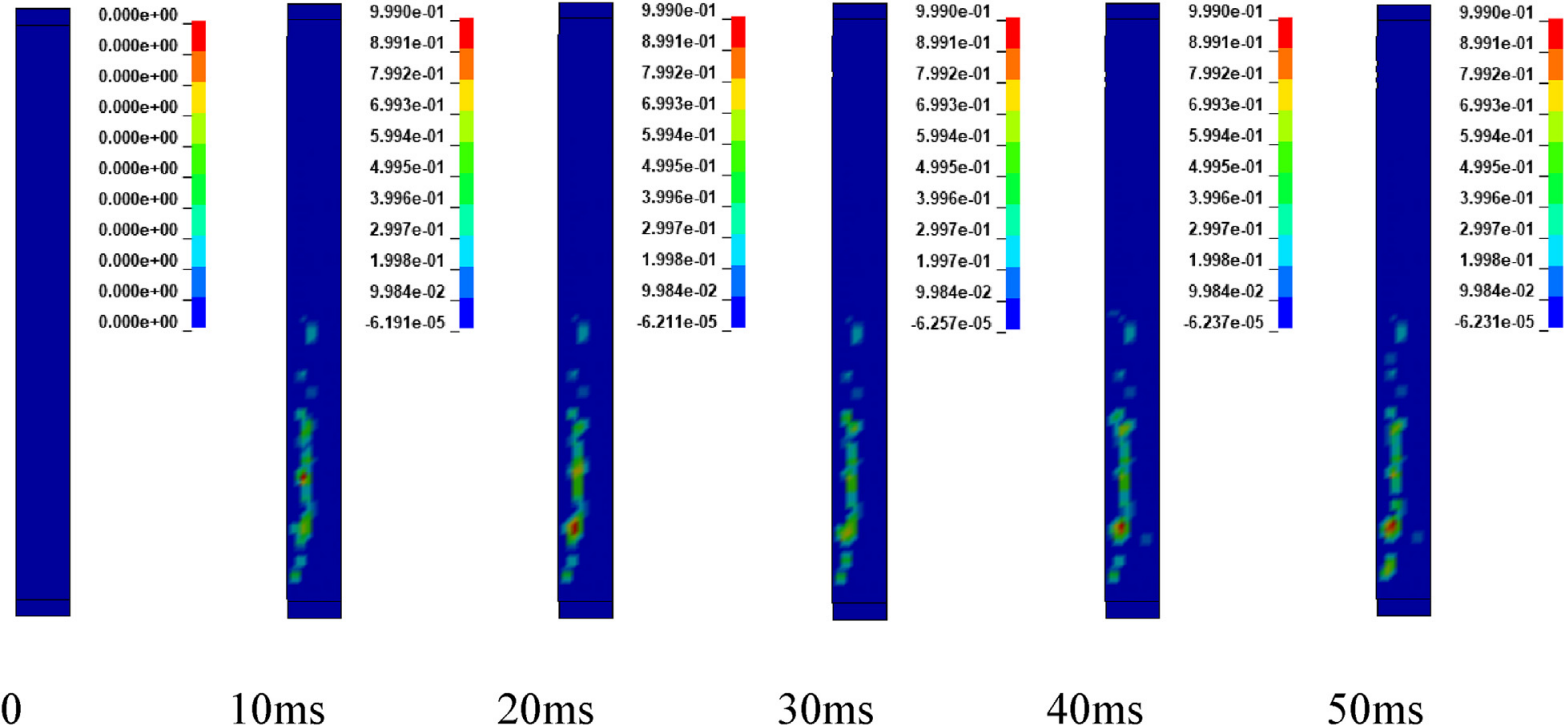
Effective plastic contour of CONV_03 RC column for blast scenario-2H.

**Figure 19:**
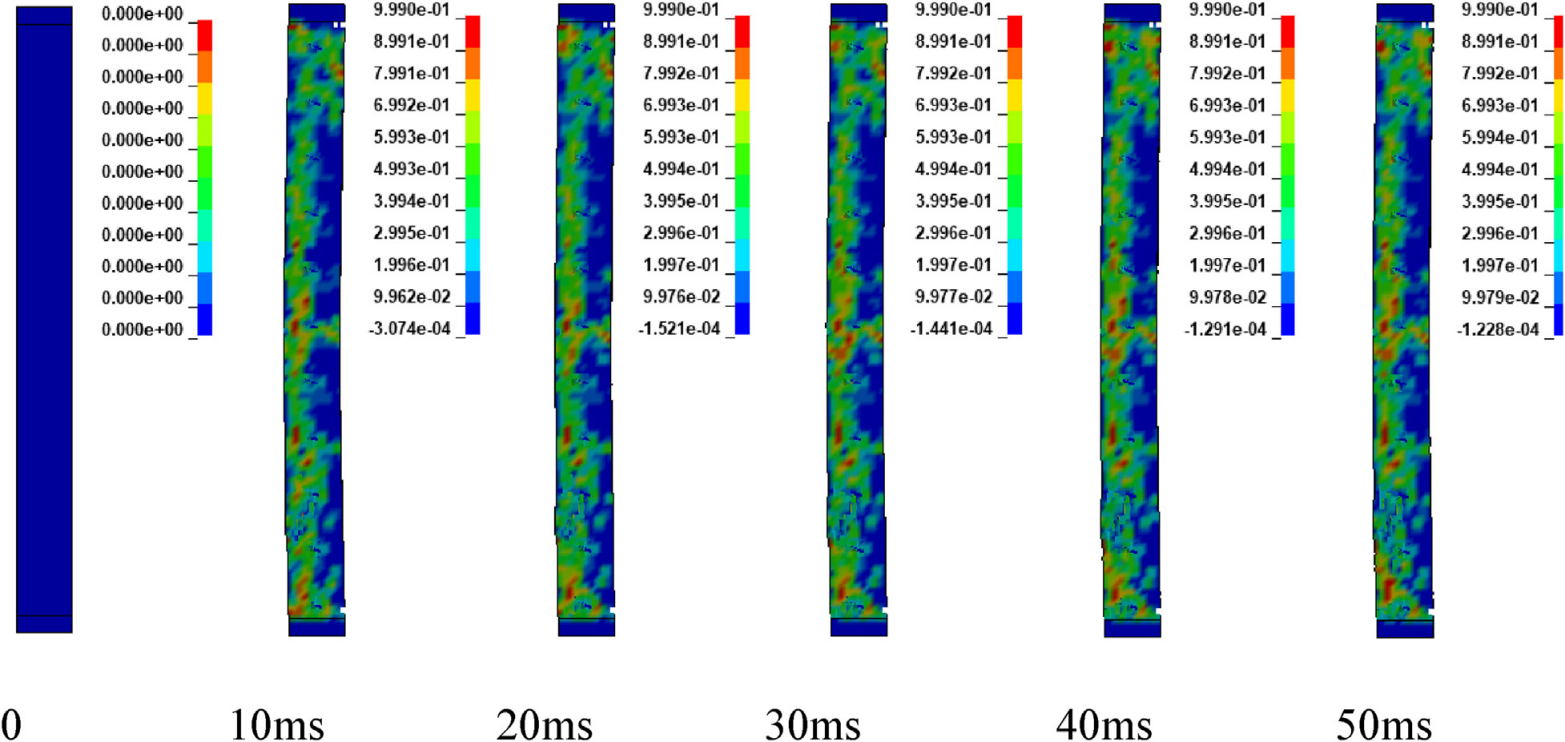
Effective plastic contour of CONV_04 RC column for blast scenario-2I.

Figures 17, 18, and 19 depicts sample damage model for CONV_02, CONV_03, and CONV_04 under blast scenario of 2G, 2H and 2I respectively. The damage threshold values were extracted from effective plastic strains fringe component module. Figure 17 represents CONV_02 column was severely damaged. Concrete in the bottom half was dilated leaving the column. Within less than 2 ms, the failure progression extends from the bottom to the top part of the column. reinforcement bars were deformed (bent) in the bottom part of the column. CONV_04 column also faces extensive damage next to CONV_02 column. Even though, fracture of concrete element and breakage of reinforcement bars were not observed, the entire column was accompanied by extensive crack which started to extend within 5 ms (see Figure 19). In contrary, CONV_03 column elucidates minor damage (almost negligible) in the bottom and completely no damage was observed in the top part of the column (see Figure 18).

## 6 Conclusions

This research study has performed FEA numerical simulations for as-built and CFRP strengthened RC columns. The numerical model developed in this study was validated with the findings of recent field controlled experimental works. Parametric studies are carried out on various charge masses, height of bursts, concrete compressive strengths, standoff distances, reinforcement detail schemes, and 0°/90° CFRP strengthened RC columns. Based on the parametric study results, the following conclusions can be drawn:

• Scaled distance parameters have significant effects on response of RC column under blast loading. Small scaled distance blast scenarios produce large lateral displacements and direct shear failures. On the other hand, lateral displacement of the RC columns decreases gradually with the increment in scaled distance.
• For small scaled distance blast scenarios, peak lateral displacements and failure modes are highly dependent on the ordinate of the vertical location of explosives (height of bursts), standoff distances, and burst types (hemispherical surface & spherical free-air bursts)
• Increasing concrete compressive strength increases column's strength and stiffness which then minimizes the peak lateral displacement of RC columns.
• Increasing 0°/90° CFRP layers had more remarkable influence for hemispherical surface bursts, lower grades of concrete and small scaled distance blast scenarios
• Compared to conventionally-detailed columns, seismically-detailed RC columns performed incredible capability to minimize peak lateral displacements and respective damages. Decreasing tie spacings are more efficient for hemispherical surface bursts and small scaled blast scenarios.
• Transverse reinforcement steel rebar schemes (arrangements) have significant effect on the lateral displacement response and failure mode of columns. Use of square-diamond shaped ties together depicts significant fall in lateral displacements and damages values for close-in blast events.

Finally, it is worthy to note that an extended parametric studies for future researches on coupled analysis of RC column using the Arbitrary Lagrangian Eulerian (ALE) method is needed.

## Declarations

### Author contribution statement

Solomon Abebe and Tesfaye Alemu Mohammed: Conceived and designed the experiments; Performed the experiments; Analyzed and interpreted the data; Contributed reagents, materials, analysis tools or data; Wrote the paper.

### Funding statement

This research did not receive any specific grant from funding agencies in the public, commercial, or not-for-profit sectors.

### Data availability statement

Data included in article/supplementary material/referenced in article.

### Declaration of interests statement

The authors declare no conflict of interest.

### Additional information

No additional information is available for this paper.
